# Supplementary material for: Complex interventions for aggressive challenging behaviour in adults with intellectual disability: A rapid realist review informed by multiple populations
Source: PLoS One. 2023 May 18;18(5):e0285590. doi: 10.1371/journal.pone.0285590 (PMC10194976; doi:10.1371/journal.pone.0285590)
Supplement: S1 File — (PDF) [file pone.0285590.s005.pdf]

Complex interventions for aggression: a rapid realist review to determine what works, for whom and in what circumstances to inform a personalised intervention for adults with learning disabilities who display aggression

Citation

Stephen Naughton, Farah Elahi, Penny Rapaport, Angela Hassiotis, Afia Ali, Maria Lahab. Complex interventions for aggression: a rapid realist review to determine what works, for whom and in what circumstances to inform a personalised intervention for adults with learning disabilities who display aggression. PROSPERO 2020 CRD42020203055 Available from: https://www.crd.york.ac.uk/prospero/display_record.php?ID=CRD42020203055

Review question

Why, for whom, and in what circumstances do complex intervention programmes for aggression work for individuals within the field of mental health (including learning disabilities); including the mechanisms of the interventions, the contexts in which they are delivered and how this impacts on outcomes?

A realist review is most appropriate because it will provide evidence on the underlying causal mechanisms and processes by which interventions operate. A realist review will also allow us to draw from other sources which are not usually included in traditional systematic reviews (such as editorials and commentaries). It will provide us with insights into underlying causal processes by which interventions can be effective. Finally, the overarching theory generated from the realist review will provide a basis for other work packages.

Searches

The following electronic bibliographic databases will be searched:

MEDLINE, PsycINFO, EMBASE, CINAHL and Health Management Information Consortium (HMIC).

Grey literature will be searched using OpenGrey.

Further information relating to the search strategy can be found in the document attached below.

Types of study to be included

Inclusion criteria: All primary and secondary research exploring complex, psychosocial intervention programmes for aggression in individuals over the age of 18 in the field of mental health.

Exclusion criteria: Studies that are not related to aggression/aggressive behaviour will be excluded. Single case designs that did not implement an experimental design will also be excluded, as well as studies that recruited participants under the age of 18 years.

Condition or domain being studied

We are conducting a rapid realist review to determine which complex, psychosocial interventions work for people with learning disabilities (and for other client groups).

Participants/population

Adults across the age range with mild, moderate, severe or profound learning disabilities (and other client groups) who exhibit aggressive behaviour in any setting.

Intervention(s), exposure(s)

This rapid realist review will review the evidence to determine which complex, psychosocial interventions work for individuals with learning disabilities (and for other client groups).

Comparator(s)/control

Not applicable.

Main outcome(s)

Severity, frequency and duration of the aggressive behaviour. An example of a validated measure that may potentially be used by studies for this outcome is the Aberrant Behavior Checklist (ABC-2), particularly the irritability subscale.

Measures of effect

Not applicable.

Additional outcome(s)

- Adaptive functioning, including communication skills.

- Mental and psychological health outcomes (such as mood and anxiety).

- Quality of life.

- Service user and carer satisfaction.

- Effects on carer stress and resilience.

- Adverse effects on other people with learning disabilities.

- Social exclusion.

- Rates of manual restraint.

- Use of psychoactive medication.

- Premature death.

- Rates of placement breakdown.

- Use of inpatient placements (including out-of-area placements).

Measures of effect

Not applicable.

Data extraction (selection and coding)

All records will be downloaded or entered by hand into an Endnote database. Duplicates will be removed by the Research Assistant (RA) and all records will be screened for eligibility according to the pre-defined inclusion and exclusion criteria.

During the initial screening of titles and abstracts, a random 5% of retrieved studies will be independently assessed by various review team members (such as Professor Angela Hassiotis, Dr Penny Rapaport etc.) This will help to ensure consistency and minimise bias. Any disagreements will be resolved through discussion.

For ease of reference and tracking, records to identify each study will be kept. It may become apparent that additional unforeseen data can be usefully charted, meaning that this is an iterative process whereby the charting table is continually updated. Key information to chart for each paper:

• Author(s).

• Study type.

• Year of publication.

• Country of origin.

• Aims/purpose.

• Study population and sample size.

• Methodology/methods.

• Intervention type/duration, comparator, outcome measures.

• Key findings that relate to the scoping review question/s.

In realist reviews, underlying mechanisms operate (or do not operate) in certain contexts to generate outcomes: Context + Mechanism = Outcome (CMO). Data will also be coded as contexts, mechanisms and outcomes. Data that informs the relationship between between these factors will be coded as CMO configurations.

Study investigators will be contacted for unreported or missing data or to seek clarification on additional details for such studies.

Risk of bias (quality) assessment

Study quality will be assessed based on relevance (whether data can contribute to theory building) and rigour (whether methods used to generate the data are credible and trustworthy), in line with the RAMESES guide for realist research (Wong et al., 2017). Studies will also be assessed for bias using tools such as the CASP (2018) tool for qualitative research.

Strategy for data synthesis

Data will be extracted for theory building in line with the research question. This will be an iterative process that will be guided by regular meetings with a Local Reference Group (LRG) and Expert Panel (EP). Theory building will be guided by the extraction of Context-Mechanism-Outcome configurations, which will seek to explain what interventions for aggression work, for whom and in what circumstances. The theories developed will be summarised and results will be reported in accordance with RAMESES-II standards for analysis and reporting, which were developed specifically for realist evaluation (Wong et al., 2017).

Analysis of subgroups or subsets

Random and fixed effects models will be used if/where possible to analyse potential subgroups. Potential subgroups that arise may include; autism spectrum disorder, gender, severity of learning disability etc.

Contact details for further information

Stephen Naughton

s.naughton@ucl.ac.uk

Organisational affiliation of the review

UCL

https://www.ucl.ac.uk/

Review team members and their organisational affiliations

Mr Stephen Naughton. UCL

Ms Farah Elahi. UCL

Dr Penny Rapaport. UCL

Professor Angela Hassiotis. UCL

Dr Afia Ali. UCL

Ms Maria Lahab. UCL

Collaborators

Professor Andre Strydom. Kings College London

Professor Umesh Chauhan. University of Lancaster

Professor Sally-Ann Cooper. University of Glasgow

Professor Richard Hastings. University of Warwick

Professor Craig Melville. University of Glasgow

Professor Andrew Jahoda. University of Glasgow

Ms Vivien Cooper. The Challenging Behaviour Foundation

Dr Laurence Taggart. Ulster University

Dr Elizabeth Steed. Queen Mary University of London

Dr Louise Marston. UCL

Dr Rashid Mansoor. UCL

Mr Brendan Leahy. Camden Disability Action

Ms Rachael Hunter. UCL

Type and method of review

Intervention, Systematic review, Other

Anticipated or actual start date

01 April 2020

Anticipated completion date

31 July 2021

Funding sources/sponsors

National Institute for Health Research (NIHR)

Grant number(s)

State the funder, grant or award number and the date of award

NIHR200120

Conflicts of interest

Language

English

Country

England, Northern Ireland, Scotland

Stage of review

Review Ongoing

Subject index terms status

Subject indexing assigned by CRD

Subject index terms

Adult; Aggression; Humans; Learning Disabilities; Research Design

Date of registration in PROSPERO

18 August 2020

Date of first submission

17 August 2020

Stage of review at time of this submission

Stage	Started	Completed
Preliminary searches	Yes	Yes
Piloting of the study selection process	No	No
Formal screening of search results against eligibility criteria	No	No
Data extraction	No	No
Risk of bias (quality) assessment	No	No
Data analysis	No	No

The record owner confirms that the information they have supplied for this submission is accurate and complete and they understand that deliberate provision of inaccurate information or omission of data may be construed as scientific misconduct.

The record owner confirms that they will update the status of the review when it is completed and will add publication details in due course.

Versions

18 August 2020
